# Supplementary material for: Effect of Intermittent Hypoxia on Metabolic Syndrome and Insulin Resistance in the General Male Population
Source: Medicina (Kaunas). 2021 Jun 29;57(7):668. doi: 10.3390/medicina57070668 (PMC8303242; doi:10.3390/medicina57070668)
Supplement: Supplementary file 1 [file medicina-57-00668-s001.zip › medicina-1235068-supplementary.pdf]

medicina

MDPI

Supplementary Table S1. Parameters of metabolic syndrome according to ODI groups.

	**ODI < 5 (*n*=66)**	**5 ≤ ODI < 15 (*n*=57)**	**ODI ≥ 15 (*n*=61)**	***p*-value**
WC*	85.16 ± 6.90	87.93 ± 6.89	91.39 ± 6.90	<0.001
SBP	110.78 ± 10.89	113.68 ± 10.84	115.25 ± 10.92	0.068
DBP*	72.85 ± 9.06	76.54 ± 9.02	77.97 ± 9.08	0.005
HDL*	54.41 ± 11.82	50.18 ± 11.77	48.35 ± 11.85	0.014
TG*	115.68 ± 69.79	137.15 ± 74.76	167.16 ± 72.78	<0.001
FBG*	99.27 ± 12.31	96.41 ± 12.26	102.95 ± 12.34	0.017
HOMA-IR*	1.43 ± 1.31	1.60 ± 1.31	2.24 ± 1.33	<0.001

WC, waist circumference; SBP, systolic blood pressure; DBP, diastolic blood pressure; .TG, triglyceride; HDL, high-density lipoprotein cholesterol; FBG, plasma fasting blood glucose; ODI, oxygen-desaturation index; HOMA-IR, homeostasis model of assessment for insulin resistance index. * indicates a p value < 0.05.
